# Multi-objective regression test suite optimization using three variants of adaptive neuro fuzzy inference system

**DOI:** 10.1371/journal.pone.0242708

**Published:** 2020-12-03

**Authors:** Ayesha Kiran, Wasi Haider Butt, Arslan Shaukat, Muhammad Umar Farooq, Urooj Fatima, Farooque Azam, Zeeshan Anwar

**Affiliations:** 1 Department of Computer & Software Engineering, College of Electrical and Mechanical Engineering, National University of Sciences and Technology (NUST), Islamabad, Pakistan; 2 Department of Computer & Software Engineering, Military College of Signals, National University of Sciences and Technology (NUST), Islamabad, Pakistan; University of Defence, SERBIA

## Abstract

In the process of software development, regression testing is one of the major activities that is done after making modifications in the current system or whenever a software system evolves. But, the test suite size increases with the addition of new test cases and it becomes in-efficient because of the occurrence of redundant, broken, and obsolete test cases. For that reason, it results in additional time and budget to run all these test cases. Many researchers have proposed computational intelligence and conventional approaches for dealing with this problem and they have achieved an optimized test suite by selecting, minimizing or reducing, and prioritizing test cases. Currently, most of these optimization approaches are single objective and static in nature. But, it is mandatory to use multi-objective dynamic approaches for optimization due to the advancements in information technology and associated market challenges. Therefore, we have proposed three variants of self-tunable Adaptive Neuro-fuzzy Inference System i.e. TLBO-ANFIS, FA-ANFIS, and HS-ANFIS, for multi-objective regression test suites optimization. Two benchmark test suites are used for evaluating the proposed ANFIS variants. The performance of proposed ANFIS variants is measured using Standard Deviation and Root Mean Square Error. A comparison of experimental results is also done with six existing methods i.e. GA-ANFIS, PSO-ANFIS, MOGA, NSGA-II, MOPSO, and TOPSIS and it is concluded that the proposed method effectively reduces the size of regression test suite without a reduction in the fault detection rate.

## Introduction

During the development lifecycle, a software system evolves several times. Software evolution occurs due to modifications in program code to add or remove a feature. A program code is generally modified more than twenty times in a minute by a Google developer [1]. It might be required to perform millions of test executions in a single day for tracking these changes which are not practically feasible. To overcome this issue, regression testing is done to make sure that these modifications have not adversely affected the older program code. Regression testing helps to detect faults and it also ensures the removal of faults in the modified version of the software system. For performing the regression testing of modified version, complete test suites that are designed to test previous releases of software along with new test cases are mostly used. But, it consumes almost 50 percent of the total cost of development and approximately 80 percent of the total testing resources [2]. Moreover, controlling the cost of executing larger test suites is a difficult task in the case of larger software and it can also affect the software quality. Test Suite Optimization (TSO) for regression testing is an active area of research and several optimization approaches have been introduced for saving the cost as well as the time of regression testing [3–7]. The existing optimization techniques mostly deal with one objective and they are static in nature i.e. do not change with changes in the system and are not real-time, hence some of the important test cases may skip out from optimized test suite [8, 9]. On the other hand, dynamic approaches are real-time and they adapt it when changes occur. Therefore, the existing single-objective static methods are not suitable for solving this problem. Consequently, it needs to be treated as a dynamic multi-objective problem that considers various alternatives for finding an optimum balance between budget as well as the effectiveness. In the literature, different techniques like Genetic Algorithms [10, 11], Ant Colony Algorithms [12, 13], Greedy Algorithms [14, 15], Fuzzy Logic [16] have been used for solving this problem.

In most of the studies, the focus is on search-based methods for optimizing the test suite for regression testing but these methods are based on discrete values for a selection of optimized test suite which may result in skipping many essential test cases [16]. The value of an objective function is computed in these techniques for searching the best solution from the sample space and the candidate solutions' value is selected if it is close to the objective function’s value. But, performing a comparison with an already defined fitness function value is not suitable in every case. For overcoming this issue and the introduction of expert judgment in test case selection, fuzzy logic is used for optimizing the test suite for regression testing [17, 18]. Fuzzy set theory has been proposed by Zadeh [19] with intention to generalize the classical notion of a set. The basic principle behind fuzzy logic is to accommodate fuzziness as a computational framework for dealing with systems which contain human language, human judgment, their behavior, emotions and decisions. The theory of fuzzy logic provides a mathematical tool to capture the uncertainties associated with linguistic and vague variables [20]. A Fuzzy Inference System (FIS) is a non-linear procedure that derives its output based on fuzzy reasoning and a set of IF-THEN rules. The FIS performs approximate reasoning like the human brain, albeit in a much more straightforward manner [21].

However, values of the parameter and the rules of the fuzzy inference system (FIS) are kept constant in the majority of existing fuzzy-based systems. These fixed values of parameters and the fuzzy rules are not applicable for every software mutant. Hence, the adjustment of parameter values is needed for every mutant. Moreover, one of the main problem faced when the model is used in real world scenarios is to train the parameter in ANFIS. Gradient Descent approaches are in the foundation of maximum ANFIS training methods, where gradient calculation in each step is tractable because many local minima may be caused by the chain rule used. Because of being local search approaches, it is difficult for gradient methods to learn parameters for global optimal model. On the other hand, designing FSs by using meta heuristics algorithms is suggested by many researchers for their ability to reduce to an optimization problem. The gradient methods are known to be local search approaches and their performances generally depend on initial values of parameters so that it is difficult for them to find the global optimal model parameters. Since the design of FSs can be reduced to an optimization problem, many researchers have proposed to design FSs by employing metaheuristics algorithms for the purpose of obtaining the global optimal solution [22].

For dealing with these issues, Adaptive Neuro-Fuzzy Inference System (ANFIS) [23] has been employed for optimizing the test suite for regression testing using four objectives i.e. rate of fault detection, coverage of requirements, time of execution and impact of requirement failure. In fuzzy systems, the introduction of expert judgment and learning abilities is done through ANFIS and a neural network based learning algorithm is used for adjustment of parameters related to membership functions and the strength of rule firing. ANFIS partially solves the issue of tuning of parameters but it is also noticeable that it is an optimization challenge to tune the ANFIS itself. To solve this problem, we have created the structure of ANFIS and presented three techniques i.e. Teaching Learning Based Optimization (TLBO), Firefly Algorithm (FA), and Harmony Search (HS), for its tuning. TLBO, FA, and HS are meta-heuristic algorithms that are utilized for solving the problem of optimization.

After a thorough literature review, we have selected these three algorithms for tuning of ANFIS. Actually, there is no need to adjust the parameters in TLBO [24]. This makes the TLBO a highly consistent optimization algorithm. It's another excellent positivity is ability to converge fast [25]. Subsequently, FA is swarm-intelligence-based, so it has the similar advantages that other swarm intelligence-based algorithms have. FA is based on attraction and attractiveness decreases with distance. This leads to the fact that the whole population can automatically subdivide into subgroups, and each group can swarm around each mode or local optimum. Among all these modes, the best global solution can be found. This automatic subdivision ability makes it particularly suitable for highly nonlinear, multimodal optimisation problems [26]. In case of HS, the implementation is easier and it is less sensitive to the chosen parameters, which means that we do not have to fine-tune these parameters to get quality solutions. Moreover, the good combination of parallelism with elitism as well as a fine balance of intensification and diversification is the key to the success of the HS algorithm [27].

These algorithms help to train the parameters of ANFIS to obtain the global optimal solution. These newly devised methods are termed as TLBO-ANFIS, FA-ANFIS, and HS-ANFIS. Optimization of regression test cases is then done with the help of tuned ANFIS. According to the experimental results, our proposed approach provides better results in comparison to the original ANFIS.

Following are the major contributions:

- For optimizing the regression test cases, we have presented three variants of self-tunable ANFIS. TLBO, FA, and HS algorithms are used for tuning of ANFIS and the produced system is thus known as TLBO-ANFIS, FA-ANFIS, and HS-ANFIS
- In terms of four selected regression test suite optimization objectives, our proposed TLBO-ANFIS, FA-ANFIS, and HS-ANFIS perform better than the existing systems

The paper is organized as follows: Section 1 includes the Introduction. Section 2 provides the Literature review. The preliminary concepts used in this paper are briefly described in Section 3, particularly the basic concept behind ANFIS, Teaching Learning Based Optimization algorithm, Harmony Search Algorithm, and Firefly Algorithm is discussed in this section. The methodology is explained in Section 4 and it consists of the system architecture and the mathematical model. In Section 5, the details about experimental setup are given; particularly, the description of case studies, analysis of techniques, and the metrics used for performance evaluation and experimental results are provided. The evaluation of proposed approach through benchmark case studies is done in Section 6. Discussion is provided in Section 7. Lastly, the conclusion of the paper is stated in Section 8.

## Previous work

In this section, we have provided an overview of state-of-the-art regression test suite optimization methods.

### Test suite optimization and greedy algorithm

Lin et al. [28] empirically evaluated three variants of the Greedy method with the help of space, siemens, gzip, and ant programs. Experimental results depict that these cost-aware methods can decrease the execution time of performing regression testing and increase fault localization capability. For minimization, Miranda and Bertolino [29] used scope-aid for boosting additional and total Greedy as well as the search and similarity centered prioritization; GE algorithm and Greedy additional selection. Several versions of *gzip*, *grep*, and *sed* is selected for performing experiments. KLEE and MILU tools are also used for determining the in-scope entities and generation of mutants. According to the results, the proposed approach can significantly reduce the test suite size without degrading the ability of fault detection. Shi et al. [30] evaluated the cost of reducing the test suite size by using actual test failures. Evaluation is done on 1478 failed builds selected from 32 GitHub projects using four different approaches i.e. Greedy, GRE, GE, and HGS. The evaluation of experimental results shows that the traditional metrics used for reducing the size of the test suite cannot properly predict the Loss in Failed-Build Detection. R Jabbarvand et al [31] presented a method for test suite minimization by employing an energy-aware coverage criterion. According to their results, the proposed Greedy based approach revealed most of the energy bugs and achieved a significant decrease in size of the test cases. Wang et al. [32] proposed a reduction approach centered on the distance to increase the efficiency of fault localization. The greedy algorithm is employed to determine the optimum solution. SIR and Siemens benchmark are used for empirical investigation and it is concluded that the proposed approach reduces the size of the test suite significantly and it provides cost and time benefits as well. Wang et al [15] argued that the improvement in the ability to localize the faults in selection of test cases can be attained with the help of an approach that considers multiple objectives at a time. They proposed the criteria to prioritize and select the test cases and used the Greedy algorithm to perform multi-objective optimization. Their approach achieved significant results in fault localization and size reduction.

### Test suite optimization and genetic algorithm

Turner et al. [33] applied Non-Dominated Sorting Genetic Algorithm II (NSGA-II) on test data of Mockito. For the selected test cases, the authors analyzed the trade-off among the time of execution and coverage of code using multiple objective-based optimization. Results depict that time of execution can be significantly reduced if a small reduction in code coverage is made. S Singhal et al [7] developed a hybrid of GA and bee colony optimization techniques known as MHBG_TCS. Time Constraint (TC) which is one of the difficult tasks in performing regression testing has been focused in this paper. The effect of variations in the value of TC is calculated in this study. According to the results of the empirical evaluation, maximum size reduction is attained beyond a few TC values. V Garousi et al. [34] introduced a variant of a Genetic algorithm for multi-objective regression test selection by considering the objective of cost as well as benefit. According to the results of the empirical evaluation, it obtained better requirement coverage and it is also cost-effective in comparison to traditional methods of test selection. Marchetto et al. [3] introduced a variant of NSGA-II, for reducing the test suites using multiple objectives, called MORE+. In this method, a reduction in the cost of execution along with requirements of application and code coverage is considered. Experimentation performed on 20 java applications show that in comparison to baseline approaches, the proposed approach provides better cost-effectiveness.

### Test suite optimization and meta-heuristic algorithms

Metwally et al. [35] presented a variant of the MFO (Moth Flame Optimization) algorithm for size reduction along with maximum coverage. Five benchmark methods are used for evaluating the performance of the proposed method. According to the evaluation results, the proposed method attains better results in comparison to the method of random generation. S Mohanty et al. [36] proposed an ant colony algorithm based regression test suite minimization approach. Three benchmark programs from the SIR repository and two own programs are used for comparison of proposed approach with four existing heuristic-based algorithms. According the experimental results, ACO based approach is capable of detecting all the faults in optimized test suite and it exhibits better time complexity as compared to other four approaches. S. R Sugave et al [4] proposed a diversity-based Dragonfly algorithm for improving the quality as well as the cost of a test suite. For achieving diversification, it used three bitwise operators. The determination of best test cases that satisfy maximum requirements is done in the proposed algorithm based on the hunting method of dragonflies. It is observed that it reduces the cost of the test suite and ensures the selection of the higher-quality test cases. S. R Sugave et al. [5] also employed two different methods to reduce the size of the test suite based on the DIV-TBAT algorithm and measure of ATAP respectively. The method based on ATAP reduces the test suite by using the Greedy algorithm. Consequently, a combination of the BAT algorithm with the mechanism of preserving diversity developed for reduction is used in the second method. It is proved from the results that diversity based BAT methods beat the classic methods in reducing the size of test cases. Choudhary et al. [37] developed a Pareto based HSA for selecting the regression test suite. Two algorithms i.e. Bat and Cuckoo Search algorithm are used for empirically evaluating the performance of the proposed technique. Results depict that the proposed method exhibits better performance as compared to these two approaches.

### Test suite optimization and fuzzy logic

Xu et al. [18] employed a Fuzzy Expert System for selecting test cases for regression testing. C language is used for creating a fuzzy expert system and data is gathered from a GSM project and 9768 test cases are used for optimization. A test plan that contained the order in which test cases need to be executed is created by a fuzzy expert system. After performing different experiments, it is analyzed that the proposed system can reduce execution time and cost associated with regression testing and it helps to find defects earlier. Haider et al. [38] used fuzzy logic to deal with the problem of optimization by considering code objective functions based on coverage. Various evolutionary algorithms are used for comparison of optimization results. It is concluded from the results that the presented approach provides adequate coverage as well as significant size and execution time reduction. For multi-objective optimization of regression test suites, Anwar et al. [39] introduced Genetic and particle swarm optimization algorithm based ANFIS and it is concluded that proposed hybrid ANFIS effectively reduces the test suite along with fault-effectiveness.

A complete description of state-of-the-art test suite optimization approaches is provided in [8].

The ANFIS model has provided good results in the case of solving various complex problems, it is judicious to use certain efficient optimizers to train its parameters (e.g. premise and consequent parameters) to improve the quality of its prediction accuracy. According to our literature review, only one study has adopted the hybrid ANFIS i.e. GA-ANFIS and PSO-ANFIS, technique for solving the problem of regression test suite optimization. As stated by Liu et al [22], the design of fuzzy systems can be reduced to an optimization problem and many researchers have proposed to design fuzzy systems by employing metaheuristics such as genetic algorithms (GAs) and particle swarm optimization (PSO). However, GAs have always been complaint about their slow convergence speed, while PSO may encounter premature convergence at the later stage of the search process and is sensitive to neighborhood topology. Moreover, Kraraboga et al [40] performed a comprehensive literature review on ANFIS training approaches and they stated that in solving the real world problems, ANFIS trained with PSO and GA provide promising results. But, new studies are required to see the impact of other algorithms in ANFIS training.

Motivated by the results discussed above, the key ultimate objective of this research is to optimize the regression test suites with the help of ANFIS tuned with three meta-heuristic algorithms.

## Preliminaries

### ANFIS

**Fig 1:**
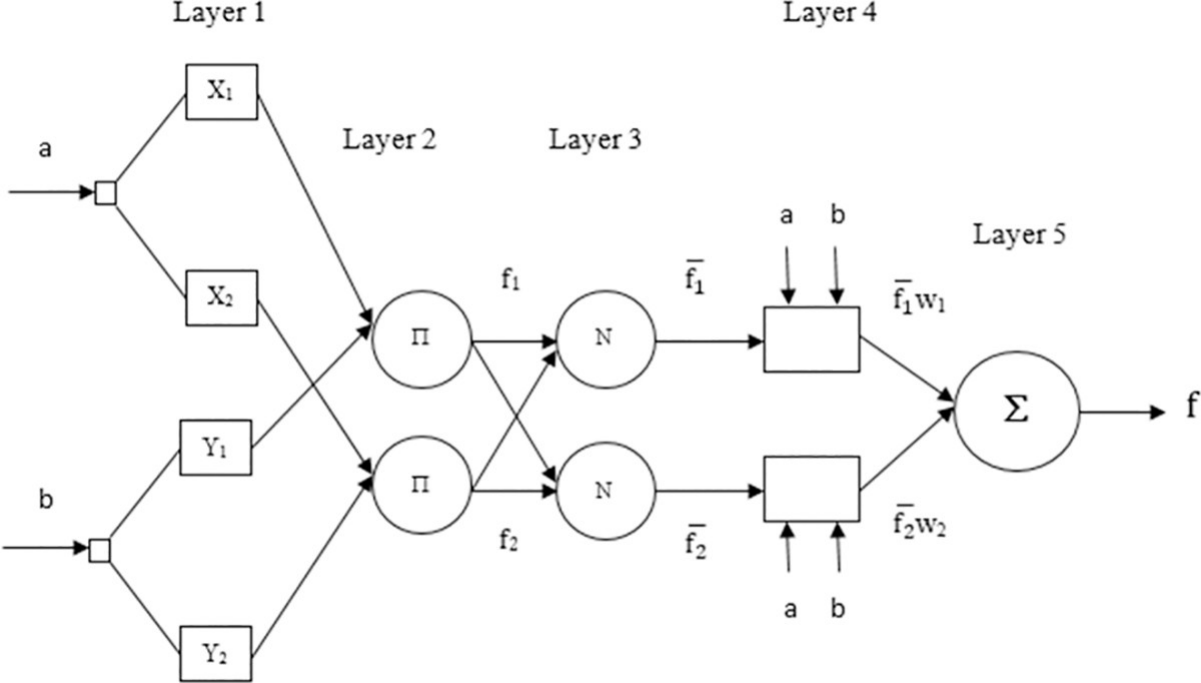
The basic architecture of ANFIS [43].

Neuro-Fuzzy Systems (NFS) are a combination of neural networks and fuzzy systems and they can be used for optimization. They also exhibit strong generalization abilities, fast and precise learning, and can easily incorporate numeric as well as linguistic knowledge for solving a problem [41]. It is also seen that ANFIS gives less Root-Mean-Square Error (RMSE) as compared to other NFS [42]. Therefore, we are using ANFIS in this research. In Fig 1, a simplified ANFIS architecture is shown that has two inputs and two if-then rules.

Rule1:IF(aisX1)AND(bisY1)THENf1=p1a+q1b+r1

Rule2:IF(aisX2)AND(bisY2)THENf2=p2a+q2b+r2

There are five layers in ANFIS and each of them provides specified functionality. In Fig 1, circles are used for the representation of adaptive nodes while the square represents the fixed nodes. The description of these five layers is given in [23]. A brief explanation is given according to which calculations are performed at each ANFIS layer using two inputs and two rules:

Layer 1: This layer has adaptive nodes and premise parameters and it consists of membership functions. It is also known as the input layer. Node functions of this layer can be determined by:

$$O_{1,i}=\mu_{x_{i}}(a)\;i=1,2$$ (1)

$$O_{1,i}=\mu_{Y_{i}}-2(b)\;i=3,4$$ (2)

a and b are given as input to node i. *Xi and Yi are the* linguistic labels (high, low, etc.) associated with this node function. Any membership function can be adopted by μ_*Ai*_ (*x*) and μ_Bi -2_ (y). If the membership function is bell-shaped, then the following equation:

$$\mu_{x_{i}}(a)=\frac{1}{1+{\left|\frac{a-z_{i}}{x_{i}}\right|}^{2y_{i}}}\;i=1,2$$ (3)

where *x*_*i*_, *y*_*i*_, *z*_*i*._ denotes the parameter set. Change in their values also causes a change in bell-shaped function.

Layer 2: This layer is associated with rules and it consists of circular nodes. This layer involves fuzzy operators; it uses the AND operator to fuzzify the inputs. The strength of firing is generated through the multiplication of all signals coming to this layer. The rule firing strength is generated as the output of this layer

$${O_{2,i}=fi=\mu }_{x_{i}}(a){.\mu }_{Y_{i}}(b)\;i=1,2$$ (4)

Layer 3: This layer has nodes that are denoted by *N* and are circular in shape. The summation of the firing strength of all rules is done at this layer. The output produced by it is known as the normalized strength of firing.

$$O_{3,i}=\bar{fi}=\frac{f_{i}}{f_{1}+f_{2}}\;i=1,2$$ (5)

Layer 4: It has square nodes that represent the input signal function. It is commonly known as the consequent layer. The output of each node in this layer is simply the product of the normalized firing strength and a first-order polynomial (for a first-order Sugeno model).

$$O_{4,i}=\bar{fi}\;w_{i}=\bar{f_{i}}(p_{i}\;a+q_{i}b+r_{i})\;i=1,2$$ (6)

This layer has consequent parameters and their set consists of {pi, qi, ri}, and these parameters are updated during the training process.

Layer 5: It is the layer that deals with the output of ANFIS. It consists of one circular node and a summation. All the incoming signals are summed up and the complete output of this ANFIS is computed by summation ∑.

$$O_{5,i}=\Sigma_{i}\bar{fi}wi=\frac{\sum_{i}f_{i}w_{i}}{\Sigma_{i}f_{i}}$$ (7)

### Teaching learning based optimization algorithm

A recently introduced population-based optimization algorithm that is inspired by the teaching and learning philosophy, is TLBO [44, 45]. At first, a population is randomly generated that represents a combination of candidate solutions. For achieving an optimal solution, a classic school learning process is simulated for modifying the feasible solution. There are two phases in it; teaching and student phase. The simulation of student learning from a teacher is done by the teaching phase. The best solution is assigned the responsibility of the teacher in this phase. By considering the present mean value of the possible solutions, the positions of other candidates' solutions are modified towards the teachers' position. In the student phase, simulation of students' learning is done by their mutual interaction. A random selection of two solutions is done during this phase. If the first randomly selected solution is better than the second one, then the second one moves in the direction of the first one. Otherwise, it moves away from the first one. The major advantage of TLBO over other optimization algorithms is that it is free of any algorithm- specific parameters tuning rather it only needs some basic parameters i.e. the total count of learners [46]. The TBLO parameters along with their assigned values are provided in Table 1.

**Table 1:** Basic parameters for TLBO.

Serial. No	Parameter	Assigned Value
1	No. of iterations	1000
2	Size of population	50

### Harmony search algorithm

Harmony search algorithm is introduced in 2001 and it has gained the attention of researchers because it provides a better trade-off in terms of exploration as well as the exploitation and it is also easy to implement. It is an evolutionary algorithm that is inspired by the music composition process of a musician. There are several possible combinations of music pitches that together make harmony and are kept in memory. Based on memory regarding rate and adjustment pitch rate, randomly generated solutions are placed directly in memory of harmony. Consequently, the calculation of pitch adjustment distance among several randomly selected solutions is done. Worst solution is then discarded and the best one is stored in harmony memory [47]. HS algorithm uses three operators to handle the exploration and exploitation and this characteristic of HS algorithm makes it unique [48]. The HS parameters along with their assigned values are provided in Table 2.

**Table 2:** Control parameters for HS.

Serial. No	Parameter	Assigned Value
1	No. of iterations	1000
2	Harmony memory size	50
3	Number of New Harmonies	20
4	Harmony Memory Consideration Rate	0.9
5	Pitch Adjustment Rate	0.1
6	Fret Width (Bandwidth)	0.02*(Var_Max_-Var_Min_)
7	Fret Width Damp Ratio	0.995

### Firefly algorithm

In 2007, Dr. Xin-She Yang developed an algorithm at Cambridge University, called Firefly Algorithm. It is amongst the latest algorithms that are inspired by nature and it is specifically based on the behavior of fireflies. The fireflies' population exhibits luminary flashing activities for performing different functions like communication, warning of predator risk, etc. This algorithm is developed by getting inspiration for these activities and under the assumption that fireflies are unisexual and their brightness level is proportional to attractiveness. Consequently, the fireflies that are less bright start moving in the direction of the brighter ones, except in the case that no firefly is brighter than other ones, at that moment it starts moving randomly [49]. Its main advantage is the fact that it uses mainly real random numbers, and it is based on the global communication among the swarming particles (i.e., the fireflies), and as a result, it seems more effective in multi-objective optimization [50]. The FA parameters along with their assigned values are provided in Table 3.

**Table 3:** Control parameters for FA.

Serial. No	Parameter	Assigned Value
1	Number of iterations	1000
2	Swarm Size	25
3	Light Absorption Coefficient (Gamma)	1
4	Attraction Coefficient Base Value	2
5	Coefficient of Mutation	0.2
6	Damping Ratio of Mutation Coefficient	0.98
7	Uniform Mutation Range	(Var_Max_-Var_Min_)* 0.05

## Methodology

**Fig 2:**
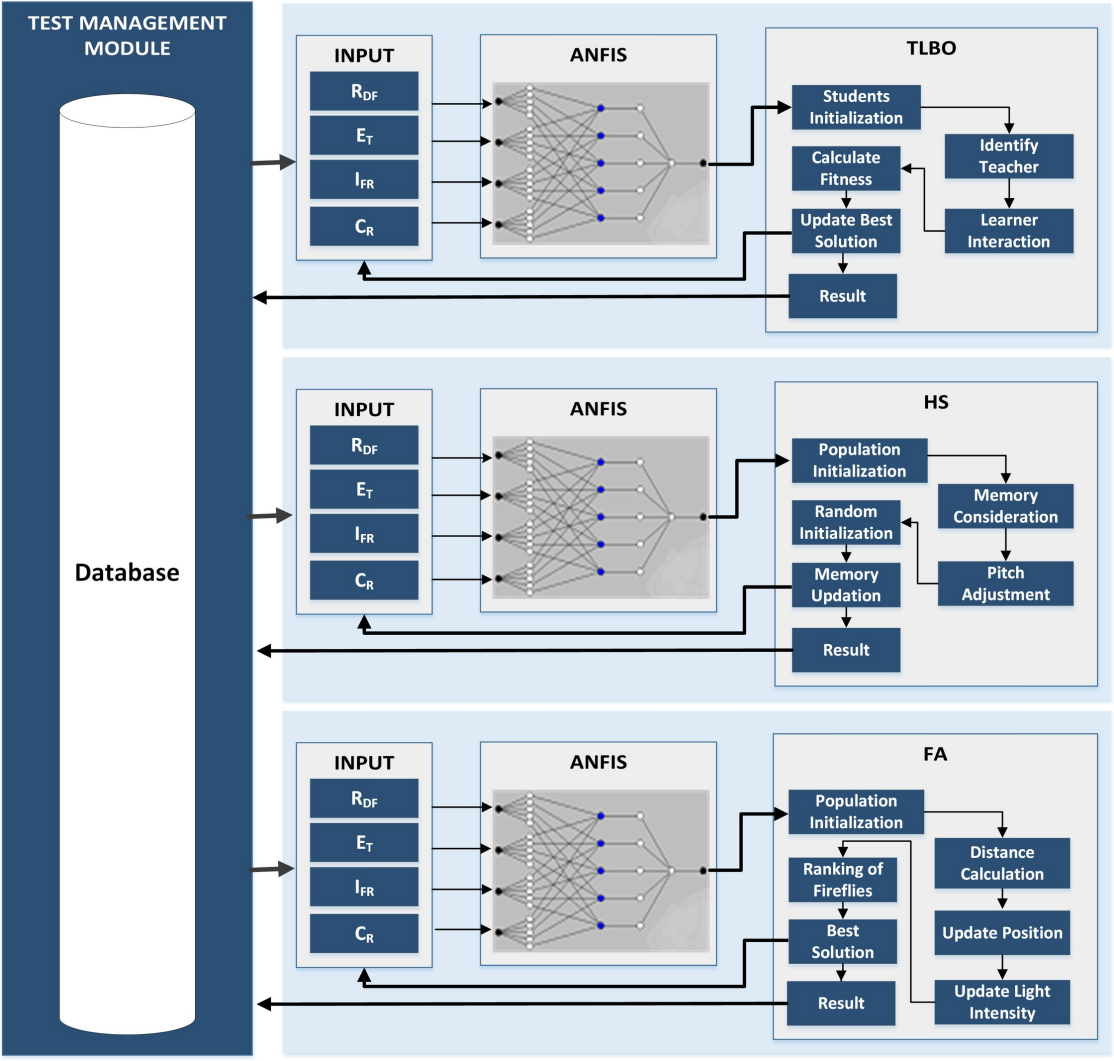
Overview of proposed methodology.

This section explains the methodology for the development of a model for optimizing the test cases for multi-objective regression testing. As presented in Fig 2, an optimizable variant of ANFIS has been devised in this research. For tuning of ANFIS, we have used the TLBO, HS, and Firefly Algorithm. The regression test suites are optimized using our proposed hybrid ANFIS. The optimization problem considered by us is to reduce the size of the test cases in such a way that they detect maximum number of faults, have minimum time of execution, covers the maximum requirements and have minimum impact of requirement failure.

### System architecture

There are two basic modules in the proposed regression test suite optimization system: 1) module for test management; 2) module for optimization. A summary of each module is given below:

#### Module for management of test cases

It has been developed for managing test cases. Test cases created by testers have been placed in it and a database has been used for saving them. This module is used for saving, modifying, and deleting test cases and generating reports. It records the execution history of the regression test suite. The execution history includes the value of time that each test case takes for the execution, their rate of fault detection, requirements that are covered by them, and the value of requirements failure impact assigned to each test case.

#### Optimization module

Execution history of test cases retrieved from the database is read by this module and the generation of data for training of ANFIS is based on this history. We have programmed three variants of ANFIS i.e. TLBO-ANFIS, HS-ANFIS, FA-ANFIS. A user can select any one of them at run time. On selection of an optimization algorithm, the ANFIS module trains the network after getting the test history and creates the FIS structure. Then, in each iteration, the selected optimization method generates multiple sets of solutions, which are values for the selected parameters of the fuzzy system. The fuzzy system is updated with each solution and then evaluated using the input training data. The evaluated output is compared with the output training data to generate the costs of the solutions. This process continues for multiple iterations until the stop condition is met, and then it returns the minimum cost solution with the optimized fuzzy system parameters. The custom model uses the fuzzy system to minimize the cost of achieving specific performance goals. The parameter solution that produces the best performance of the custom model is returned as the optimization result. Finally, it displays the results and errors of training and testing data.

### Mathematical model

In Table 4, we have provided the notations that are used for defining the problem of regression test suite optimization and they are also used in different equations defined in this article. Test cases for regression testing that are present in a test suite are denoted by S_C_ and they are collectively represented as test suite *O*. It is required to optimize test suite *O*_*T*_ in such a way that *O*_*T*_ belongs to *O* and the size of *O*_*T*_ is less than *O*. It can be mathematically represented as:

$$O_{T}\subseteq O\;and\;|O_{T}|<|O|$$ (8)

**Table 4:** Variables.

Serial. No	Parameter	Notation
1	Test Case	S_C_
2	Test Suite	O
3	Optimized Test Cases	O_T_
4	Coverage of Requirements	C_R_
5	Impact of Requirement Failure	I_FR_
6	Total requirements in a test suite	T_R_
7	Requirements covered by a test case	T_C_
8	Rate of Detected Faults	R_DF_
9	Total faults in a test suite	T_F_
10	Faults detected by a test case	D_F_
11	Time of Execution	E_T_

We have selected four objectives for optimizing the test suites for regression testing i.e. rate of fault detection, time of execution, coverage of requirements, and impact of requirement failure. These objective functions are selected after a thorough literature review.

Most of the optimization techniques for regression test suite are coverage based, therefore, we have used requirement based technique for covering black-box testing that is very rare in literature. Moreover, only one study is available that has used ANFIS for dealing with multi-objective regression test suite optimization problem. In order to compare and validate the effectiveness of our proposed ANFIS based approach, we have used the same objectives. The objectives selected for optimization are presented in the form of variables. The calculation is done for the input variables and they are given as input to the ANFIS. The description of all these variables is given below:

1. Fault Detection Rate (R_DF_) defines how many faults are detected by each test case. The formula given below is used for calculating it:
  $$R_{DF}=\frac{D_{F}}{T_{F}}$$ (9)
2. Requirement Coverage (C_R_) depicts a count of requirements that have been covered by a test case. We used the formula given below for measuring it:
  $$C_{R}=\frac{T_{C}}{T_{R}}$$ (10)
3. Execution Time (E_T_) represents the time a test case takes for execution. The execution time of different test cases is measured with the help of a timer function.
4. Requirement Failure Impact (R_FD_) is a parameter of reliability and according to the importance/criticality as well as the fault revealing ability, it is assigned to each requirement by the testing experts. During the phase of requirement gathering, it could be allocated to requirements that are critical as they are necessary to be thoroughly checked in every test suite. Each test case has some associated value of requirement coverage and based on this value, RFD is assigned to each test case. The range of the value for RFD lies between 0–1.

The final objective function considered by us is the selection of *O*_*T*_ which has a maximum rate of fault detection, minimum time of execution, covers the maximum requirements, and has a minimum impact of requirement failure. This function can be depicted as:

$$F={}_{j=1}^{n}\prod [Max({RDF}_{j})+Max({CR}_{j})+Max({IFR}_{j})+Min({ET}_{j})]$$ (11)

The Fuzzy sets are given as input to ANFIS and they include variables of the semantic type e.g. High, Medium, and Low. Following are the fuzzy-based sets that are chosen for optimization of test cases for regression testing:

$$R_{DF}=\{H,M,L\}$$ (12)

$$E_{T}=\{H,M,L\}$$ (13)

$$C_{R}=\{H,M,L\}$$ (14)

where High, Medium, and Low are represented by *H*, *M*, and *L* respectively.

$$I_{FR}=\{C,M,N\}$$ (15)

where Critical, Medium, and Normal, are represented by *C*, *M*, and *N* respectively.

There is only one output variable and it represents the fitness of each test case to be included or discarded from the list of optimized ones.

## Experiments and results

For implementing our proposed approach and comparing it with the selected Computational Intelligence (CI) based methods, we have performed different experiments. Two case studies i.e. S1 and S2 Datas are selected for this purpose and the focus of all these experiments is on the reduction of regression test cases. The implementation of ANFIS using three different optimization algorithms is done on each case study. In this research, we have used MATLAB R2016a for implementing our proposed methods. It is always required to take the average of multiple runs as a result of soft computing algorithms. Therefore, we have taken the average of multiple runs for each variant of ANFIS.

### Case studies

For evaluating and performing a comparison of our approach with other approaches that have already been implemented, we have selected the Previous Date Problem (PDP) as our first case study. A complete description of the program and information related to testing this program is available in [51]. C language is used for the implementation of PDP and the creation of test cases is done with the help of Equivalent Class Partitioning (ECP) and Boundary Value Analysis (BVA) and methods. 33 test cases have been developed for this program. Faults Seeding is employed for the insertion of 6 faults in the original program.

For performing controlled experimentation on software testing, a lexical analyzer namely Siemens Print Tokens (SPT) [52] is developed in C Language and this is taken as our Case Study II. There are 539 LOC, 18 functions 7 seeded errors in SPT code. It consists of 4130 test cases. The code, test cases, and faulty versions of SPT can be downloaded from SIR. The execution time of all test cases is measured with the help of a timer function, and the universal test script provided by SIR is used for measuring the rate of fault detection. Both datasets have been used in literature for optimizing the regression test suites [17, 31].

### Analysis of techniques

**Fig 3:**
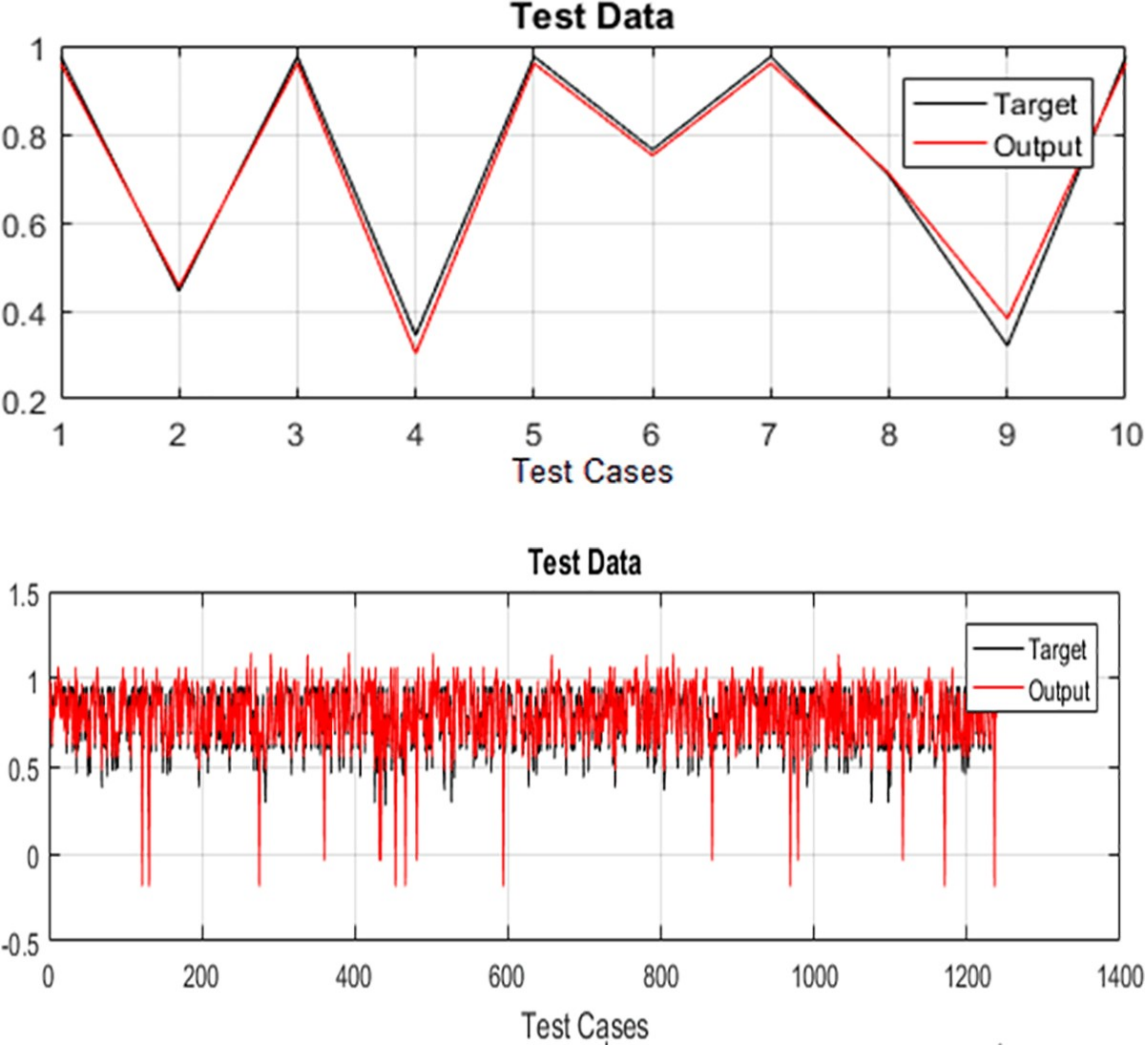
Graphical representation of difference between target and output by implementing TLBO-ANFIS (A) for PDP (B) for SPT.

**Fig 4:**
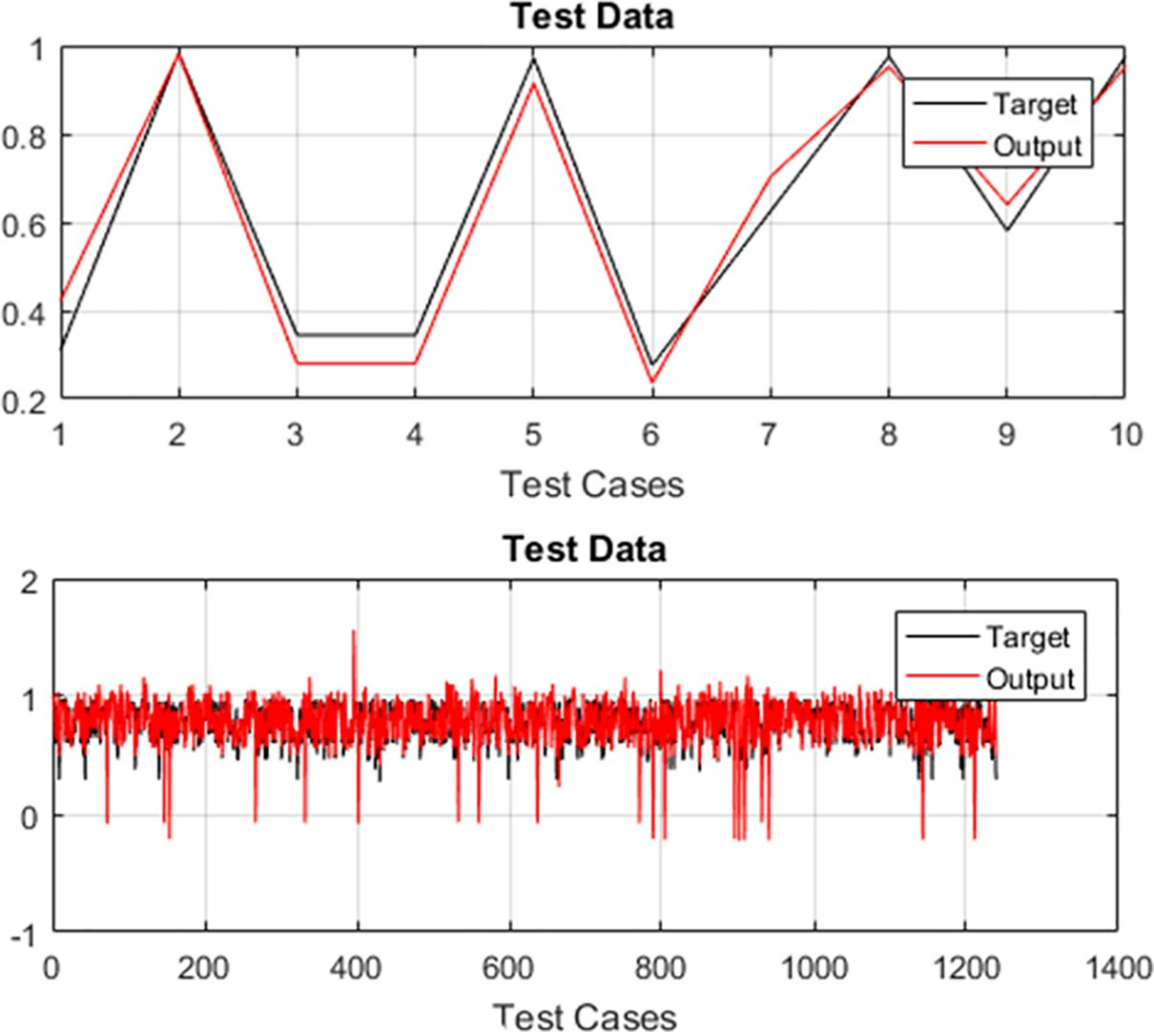
Graphical representation of difference between target and output by implementing HS-ANFIS (A) for PDP (B) for SPT.

**Fig 5:**
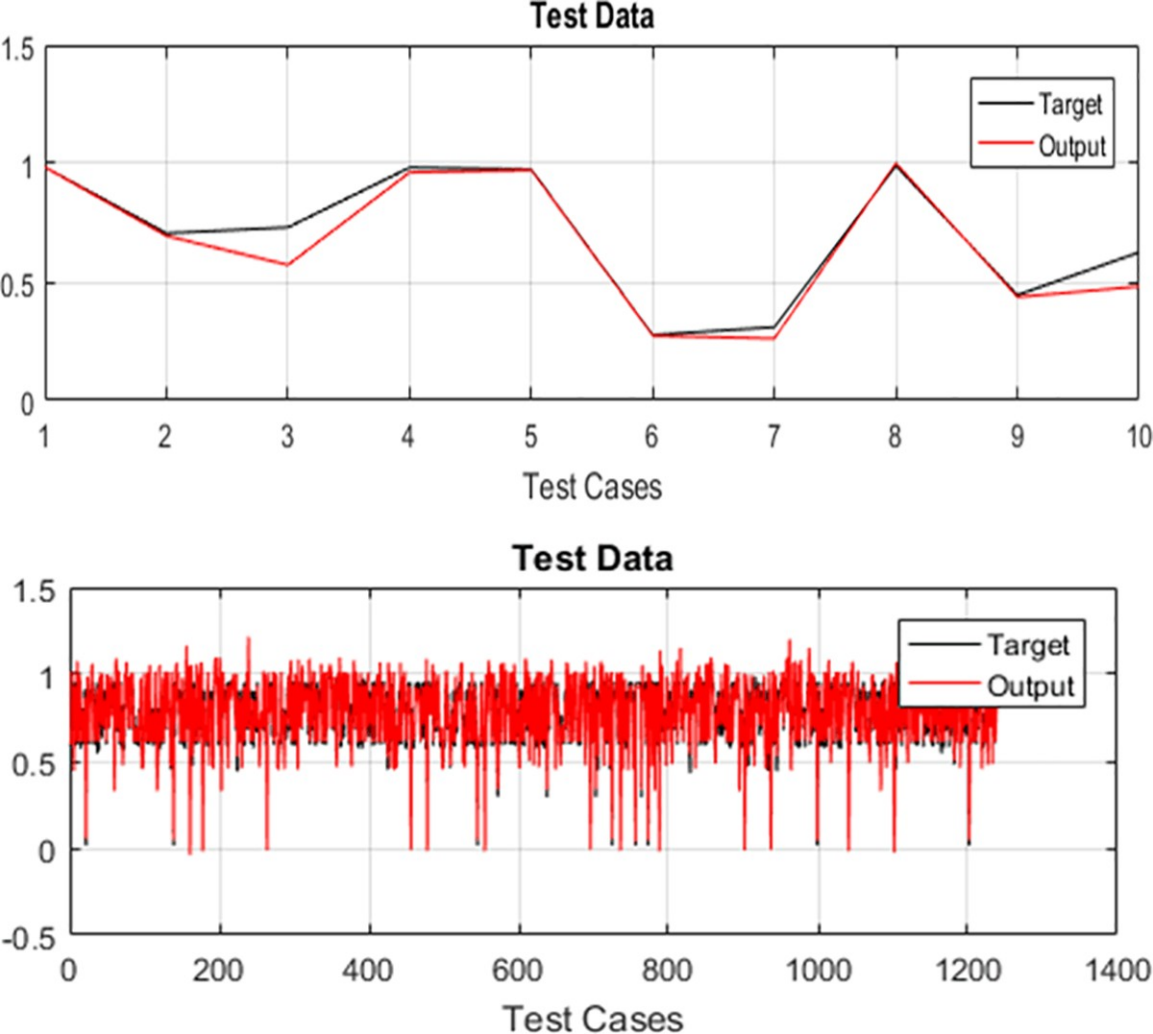
Graphical representation of difference between target and output by implementing FA-ANFIS (A) for PDP (B) for SPT.

The test cases of both case studies are executed and their test history is recorded for optimizing the regression test suites. For training the selected variant of ANFIS, 70% randomly selected test cases are used while testing is done on the remaining 30% randomly selected test data. The test data plots for both case studies are used for evaluation. Plots of target and output of PDP and SPT for TLBO-ANFIS, HS-ANFIS, and FA-ANFIS are given in Figs 3–5 respectively.

In the literature, researchers have used “*Standard Deviation*” (SD) and “*Root Mean Square Error*” (RMSE) for measuring the performance of ANFIS [39]. Moreover, according to Precup et al. [53], the RMSE is viewed as a global performance index. Therefore, we have also used these two metrics for evaluating the performance of proposed ANFIS variants. The target values for both case studies are generated using the method provided in [54]. The error of the system is calculated by finding the variance among target value and the output value generated by ANFIS variants. The calculated values of *RMSE* and *SD* for both case studies are provided in Tables 5 and 6. From Figs 3–5, it can be seen that TLBO-ANFIS accurately predicts the target values for PDP as compared to two other two variants. For SPT, the RMSE range for TLBO-ANFIS is between 0.005 and 0.061 and the error is between 0.007 and 0.050 for most of the test cases. For HS-ANFIS and FA-ANFIS, the range of RMSE is between 0.007 and 0.073, and 0.010 and 0.048 respectively. Therefore, it is evident that ANFIS-TLBO has fewer prediction errors as compared to ANFIS-HS and ANFIS-FA. Moreover, less variation in system and target value is observed by implementing TLBO-ANFIS. In Table 7, we have also provided the performance comparison of proposed ANFIS variants in terms of their execution time.

**Table 5:** Prediction error of proposed ANFIS variants on first case study.

Case Study I: PDP						
Error	TLBO-ANFIS		HS-ANFIS		FA-ANFIS	
	Training	Testing	Training	Testing	Training	Testing
**SD**	0.05	0.04	0.07	0.08	0.04	0.04
**RMSE**	0.05	0.06	0.06	0.06	0.07	0.07

**Table 6:** Prediction error of proposed ANFIS variants on second case study.

Case Study II: SPT						
Error	TLBO-ANFIS		HS-ANFIS		FA-ANFIS	
	Training	Testing	Training	Testing	Training	Testing
**SD**	0.05	0.05	0.06	0.06	0.04	0.04
**RMSE**	0.05	0.05	0.06	0.07	0.04	0.04

**Table 7:** Comparison of proposed ANFIS variants in terms of execution time.

	TLBO-ANFIS	HS-ANFIS	FA-ANFIS
**Execution Time for Previous Date Problem**	1.71 min	1.93 min	4.32 min
**Execution Time for Siemens Print Token**	6.89 min	4.68 min	38.71 min

### Metrics for performance evaluation

We analyzed the results of ANFIS based approaches for optimizing the regression test cases in terms of four metrics. A brief explanation of these metrics is given below.

1 Size Reduction

Categorization of the suitability of a test case is done for achieving a reduction in size when the ANFIS generates the output of optimization. Only those test cases are chosen that exhibit high suitability value. The calculation of the reduction percentage of the test suite has been done by the following formula:

$$RTS=\frac{\left|O|-|O_{T}\right|}{\left|O\right|}*100$$ (16)

where *RTS* represents the percentage reduction in test suite size, *O* represents the original test suite and *O*_*T*_ represents the test suite after optimization

2 Loss in Rate of Detection of Faults

Unsuitable test cases are eliminated from the optimized test suite are hence the size of reduces and it may also cause a decrease in the fault detection rate of the test suite. The following formula has been used for calculating percentage faults detection loss:

$$FDR=\frac{R_{DF}-R_{DF}\prime }{R_{DF}}*100$$ (17)

where FDR denotes percentage loss in the rate of fault detection *R*_*DF*_ represents the original test suites’ fault detection rate and *R*_*DF*_*’* represents the fault detection rate of test suite after optimization.

3 Reduction in Time of Test Suite Execution

The following formula has been used for calculating the reduction in execution time:

$$RTE=\frac{E_{T}-E_{T}\prime }{E_{T}}*100$$ (18)

where RTE denotes percentage reduction in time of execution, *E*_*T*_ represents the execution time of original test suite and *E*_*T*_*’* represents the execution time of optimized test suite

4 Reduction in Coverage of Requirements

Elimination of test cases can also cause a loss in coverage of requirements. The formula given below is used for calculation of the reduction in requirement coverage after optimization of test cases:

$$LRC=\frac{C_{R}-C_{R}\prime }{C_{R}}*100$$ (19)

where LRC denotes percentage loss in coverage of requirement, *C*_*R*_ represents the requirement coverage of original test suites, and *C*_*R*_*’* represents the requirement coverage of test suite after optimization.

### Experimental results

As shown in Table 8, the results of PDP in terms of RTS, FDR, RTE, and LRC by implementing TLBO-ANFIS, HS-ANFIS, and FA-ANFIS are provided. Due to elimination of un-suitable test cases, the size of test suite is reduced and it may also cause a decrease in the fault detection rate of the optimized test suite.

**Table 8:** Evaluation of experimental results.

Case Study I: PDP					Case Study II: SPT			
Algorithm	RTS	FDR	RTE	LRC	RTS	FDR	RTE	LRC
**TLBO**	57.57	0	65.19	59.48	54.54	0	55.52	48.01
**HS**	60.60	0	63.52	62.71	57.57	0	62.47	53.39
**FA**	63.63	25	58.14	64.95	67.67	0	69.20	72.11

It can be seen that for the first case study, both TLBO-ANFIS and HS-ANFIS provide a significant reduction in the time of executing test cases, and they almost take equal time for their execution as shown in Table 7. But, the coverage of requirement by implementing TLBO-ANFIS is higher as compared to the other two variants. For SPT, FA provides the highest reduction in terms of size but it also has a maximum reduction in requirement coverage. On the other hand, TLBO and HS provide good trade-off in terms of four optimization objectives. From these results, it can be concluded that the optimization of the regression test suite using TLBO-ANFIS provides the best results because it gives better requirement coverage as compared to the other two approaches. In the same way, HS-ANFIS and ANFIS-FA significantly decrease the size of the larger test data i.e. Siemens Print Token. But, they provide lesser requirement coverage as compared to TLBO-ANFIS. It must be noted that the time of executing FA-ANFIS is quite higher than the other two variants as depicted in Table 6. Hence, it is entirely dependent on the specified optimization objective that what variant of ANFIS must be chosen. If the goal is to achieve maximum reduction with a 100% rate of faults detection, then FA-ANFIS may be used but if one wants to achieve maximum requirement coverage along with detection of all faults then TLBO-ANFIS is the best choice.

## Benchmarking and validation

This section deals with the comparison of proposed ANFIS based approach with state-of-the-art optimization approaches used by researchers for performing regression testing. Six optimization approaches are selected for comparison after careful analysis and a thorough literature review. The approaches include ANFIS-GA, ANFIS-PSO, Multi-Objective Genetic Algorithm (MOGA), Multi-Objective Particle Swarm algorithm (MOPSO), Non-dominating Sorting Genetic Algorithm (NSGA-II), and TOPSIS. The results of these approaches are taken from already published papers that have used the same optimization objectives for regression test suite optimization [17, 39]. The criteria of evaluation are RTS, FDR, RTE, and LRC. A detailed description of these four evaluation metrics is already provided in Section 5.3.

### Case study-I

**Fig 6:**
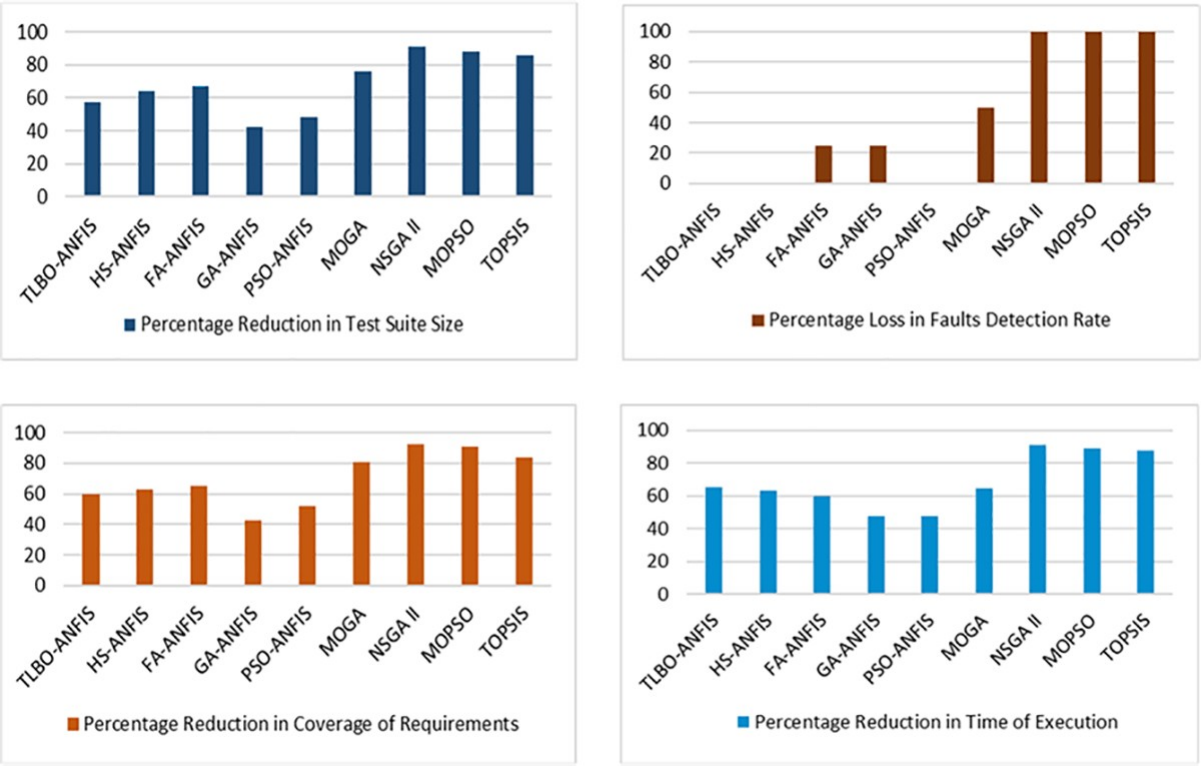
Comparison results for PDP.

It has been noted that the size of regression test cases can significantly reduce using NSGA-II, TOPSIS, and MOPSO methods. But, the main disadvantage is that this size reduction also causes a decrease in the rate of detected faults which is against our optimization objective. The requirement coverage of the test suite also decreases by employing these techniques. The comparison results of PDP in terms of the four optimization objectives are given in Fig 6. The major advantage of TLBO over the other two variants is that it overcomes some innate weaknesses of other optimization algorithms like the tuning of parameters. It does not require any specific controlling parameter as discussed in Section III B. The comparison of our proposed ANFIS variants with the latest optimization methods reveals that TLBO-ANFIS provides the most effective results for optimizing the regression test suite of PDP as it finds a better trade-off among our four optimization objectives. ANFIS trained with TLBO reduces the size of regression test cases to 57.57% without degrading the effectiveness of fault detection. This technique also causes a reduction in the time of executing test cases up to 65.19% and the Requirement coverage of TLBO-ANFIS is also higher than HS-ANFIS and FA-ANFIS.

### Case study-II

**Fig 7:**
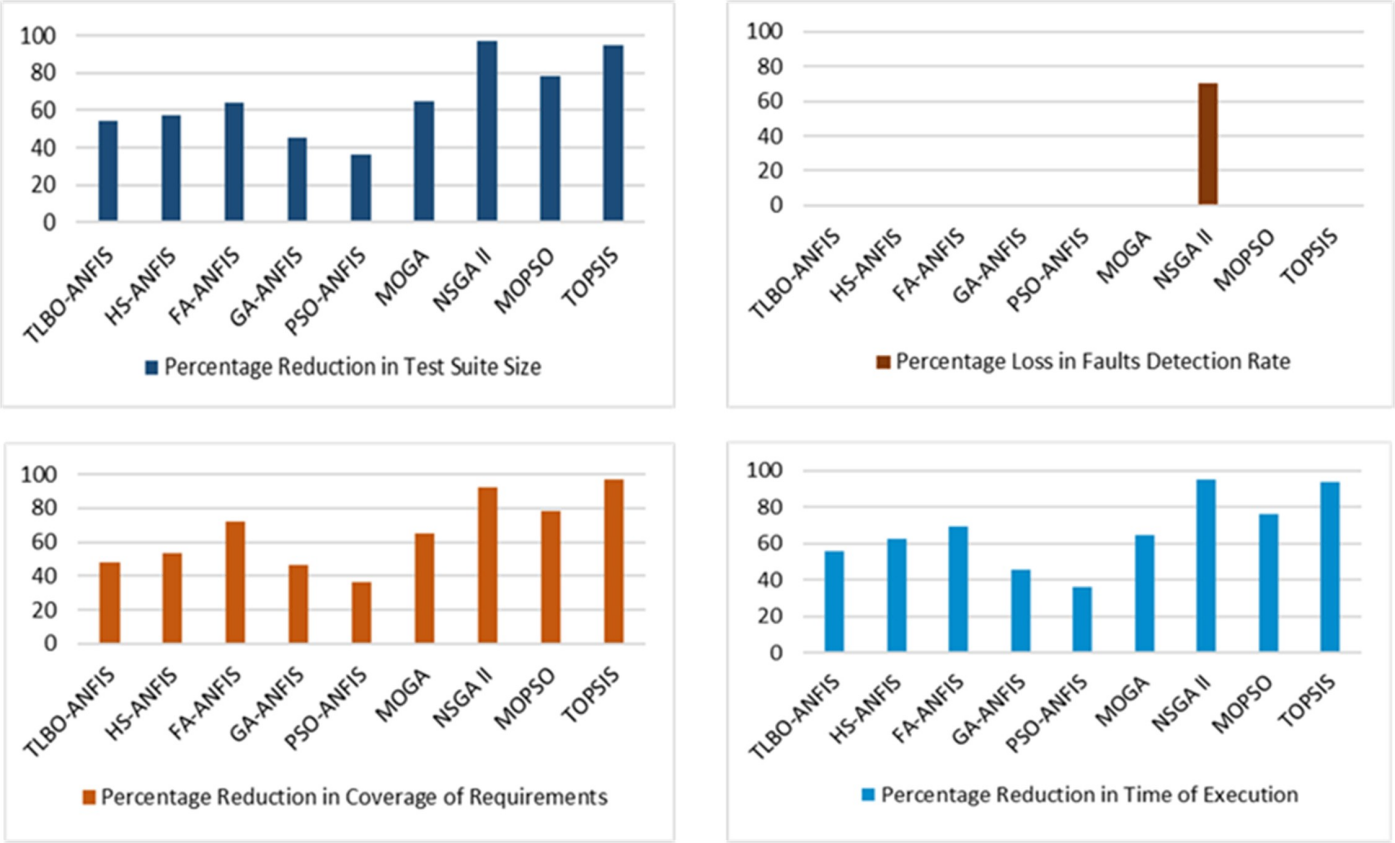
Comparison results for SPT.

According to the results of experiments performed on another benchmark test suite i.e. Siemens Print Tokens, it is revealed that almost 90% reduction in the test suite is achieved by employing NSGA-II and TOPSIS. But, it is not suitable to use NSGA-II for optimizing the test suites for regression testing because it can only detect 30% of faults. On the other hand, the remaining optimization approaches are capable of detecting all faults. Hence, other criteria such as coverage of requirements, test suite size reduction can be used for selecting the appropriate optimization method. The optimization approach which finds the best trade-off, i.e., capable of reducing the size of the test suite and detects all the faults along with higher coverage of requirements is the best candidate for selection. The requirement coverage of TLBO-ANFIS is greater than that of the other two variants. By comprehensively analyzing the results of our proposed variants of ANFIS i.e. HS-ANFIS, TLBO-ANFIS, and FA-ANFIS, it is revealed that TLBO-ANFIS and FA-ANFIS can detect 100% faults but TLBO-ANFIS provides better coverage of requirements as compared to FA-ANFIS. Therefore, it is safe to use TLBO-ANFIS because of its higher requirement coverage. The comparison results for SPT are presented in Fig 7.

## Discussion

This article introduces three variants of ANFIS i.e. TLBO-ANFIS, FA-ANFIS, and HS-ANFIS for regression test suite optimization. Many researchers have proposed to design fuzzy systems by using GAs and PSO. However, GAs have slow convergence speed, while PSO is sensitive to neighborhood topology and at the later stage of the search process it sometimes encounter premature convergence and. The performance evaluation of proposed ANFIS variants is done through two benchmark case studies i.e. Previous Date Problem and Siemens Print Tokens. In this context, it can be said that the size of case studies is slightly small to perform a realistic evaluation. We selected Previous Date Problem and Siemens Print Tokens case studies because they are used as a benchmark in literature. Here, the objective is to demonstrate the effectiveness of proposed methods and it is fairly achieved through given case studies.

We have employed four objectives for optimization i.e., rate of detection of faults, time of executing test cases, coverage of requirements, and impact of requirement failure to select regression test suite. The selection of those test cases is done that has a maximum rate of fault detection and maximum coverage of requirements. For implementing our proposed approach and comparing it with selected CI-based methods, we have performed different experiments. According to the experimental results, the proposed TLBO-ANFIS approach provides best results in terms of four selected optimization objectives. Although ANFIS-HS and ANFIS-FA significantly reduce the test suite size along with execution time of test cases, but they also cause a decrease in coverage of requirements as compared to TLBO-ANFIS. Moreover, the execution time of FA-ANFIS is quite high for the second case study. The results, as given in Tables 5–7, prove that the proposed TLBO-ANFIS variant provides better results as compared to other two variants and is capable of safely optimizing the regression test suites.

To get effective results, it is mandatory to have an accurate measurement of these objectives. Six optimization approaches are selected for comparison after careful analysis and the approaches include ANFIS-GA, ANFIS-PSO, Multi-Objective Genetic Algorithm (MOGA), Multi-Objective Particle Swarm algorithm (MOPSO), Non-dominating Sorting Genetic Algorithm (NSGA-II), and TOPSIS. Although the reduction in size is directly proportional to the reduction in requirement coverage but according to our experimental results, TLBO-ANFIS covers almost 7% more requirement as compared to the reduction in size. Additionally, the requirement coverage reduction of TLBO-ANFIS is quite similar to GA-ANFIS but it provides almost 9% more reduction in size with only a difference of 0.03 in terms of RMSE. Hence, it can be said that the proposed TLBO-ANFIS presents better trade-off in terms of size reduction and requirement coverage reduction as compared to other methods. Moreover, not even a single study is available in the literature that has used TLBO, HS, or FA for tuning of ANFIS for optimizing test suites for regression testing.

Following are the key contributions of the proposed ANFIS based method:

- Cost-effectiveness: Our proposed approach is automated and it performs automated analysis for selecting the optimized test suites, hence the cost is also low as it is done by the computer. Furthermore, sophisticated servers are not required for implementing these techniques
- Free of parameter tuning: All of the evolutionary and swarm intelligence-based algorithms are probabilistic algorithms and require common controlling parameters, like the population size, number of generations, elite size, etc. In addition to the common control parameters, algorithm-specific control-parameters are required. For example, GA uses the mutation rate and crossover rate. Similarly, PSO uses the inertia weight, as well as social and cognitive parameters. The proper tuning of algorithm-specific parameters is a very crucial factor that, affects the performance of the above-mentioned algorithms. The improper tuning of algorithm-specific parameters either increases the computational effort or yields a locally optimal solution. Unlike these intelligent optimization techniques, any parameter adjustment is not required for TLBO. Our proposed TLBO-ANFIS does not require any tuning of parameter and it also provides better results as compared to other variants of ANFIS on the selected optimization problem

## Conclusion

In this article, we have proposed and implemented three variants of ANFIS i.e. TLBO-ANFIS, HS-ANFIS, and FA-ANFIS using four objectives for optimization of regression test suites. A comparative analysis of these techniques is done with the latest optimization methods for validating the results. After performing experiments on two benchmark case studies, it is revealed that all of these three variants are capable of detecting all the seeded faults for larger test data. It is also evident from the experimental results that HS-ANFIS and FA-ANFIS significantly reduce the test suite size along with the execution time of the test cases, but they also cause a decrease in the coverage of requirement and the execution time of FA-ANFIS is quite high. However, in comparison to the other two variants, TLBO-ANFIS gives better results in terms of requirement coverage. The key advantage of TLBO-ANFIS over the other two variants is that it is free of any parameter tuning. According to our experimental results, the proposed techniques i.e. TLBO-ANFIS, HS-ANFIS, and FA-ANFIS can effectively detect the seeded faults but overall TLBO-ANFIS provide better results. Thus, our proposed variants of ANFIS are capable of safely optimizing the test suite for regression testing.

In the future, work can be done on increasing the coverage of requirement along with the maximum reduction in the size of test suite as they are directly dependent on each other. For comparing the experimental and industrial results, the application and validation of proposed approach can be done on industrial projects.

## Supporting information

S1 Data Case study 1. (XLSX)

S2 Data Case study 2. (XLS)
